# Longitudinal OCT changes of the peripapillary RNFL after different glaucoma interventions – a comparative study

**DOI:** 10.1186/s12886-026-05160-6

**Published:** 2026-07-31

**Authors:** Carlo Fiore, Xiao Shang, Nathanael Urs Häner, Joel-Benjamin Lincke, Martin Sebastian Zinkernagel, Jan Darius Unterlauft

**Affiliations:** 1 Department of Ophthalmology, Bern University Hospital Inselspital, University of Bern, Freiburgstrasse, Bern, 3010 Switzerland; 2 Department of Ophthalmology, University of Leipzig, Liebigstrasse 12, 04103 Leipzig, Germany

**Keywords:** Glaucoma, Trabeculectomy, Deep sclerectomy, XEN, Preserflo, Retinal nerve fiber layer

## Abstract

**Background:**

The study’s objective was to investigate the development of the peripapillary retinal nerve fiber layer (pRNFL) thickness after Deep Sclerectomy (DS), XEN microstent implantation and Preserflo (PF) microshunt implantation compared to Trabeculectomy (TE).

**Methods:**

This retrospective monocentric study analyzed eyes with primary open-angle glaucoma (POAG) after TE, DS, XEN- or PF-implantation over 2 years. Intraocular pressure (IOP), number of IOP-lowering medications, best-corrected visual acuity (BCVA), and success rates were analyzed. pRNFL was measured by spectral domain optical coherence tomography (SD-OCT).

**Results:**

462 eyes were analyzed. 108 underwent TE, 183 DS, 90 XEN- and 81 PF-implantation. Mean IOP and mean number of IOP-lowering medications decreased, while BCVA remained stable in all groups. Mean pRNFL significantly decreased in all groups, changing from 64.6 ± 2.0 to 57.8 ± 1.9 μm (-6.8 μm, *p* < 0.001), 63.6 ± 1.8 to 58.3 ± 2.5 μm (-5.3 μm, *p* = 0.005), 63.2 ± 2.2 to 60.0 ± 2.1 μm (-3.2 μm, *p* = 0.008) and 62.1 ± 1.8 to 59.9 ± 1.8 μm (-2.2 μm, *p* = 0.008) in the TE, DS, XEN and PF groups. Between 1 and 2 years postoperatively, no further statistically significant decline was observed in any group (TE: *p* = 0.131; DS: *p* = 0.374; XEN: *p* = 0.129; PF: *p* = 0.471).

**Conclusions:**

Despite success in reducing IOP and IOP-lowering medications, the pRNFL declines during the first postoperative year, with no further statistically significant decline observed during the second year.

**Supplementary Information:**

The online version contains supplementary material available at 10.1186/s12886-026-05160-6.

## Introduction

The glaucomas are a group of eye diseases that lead to demise of the retinal ganglion cells (RGC) [1] and therefore to the development of visual field defects and blindness when left insufficiently treated [2]. Loss of RGC and their axons is indirectly measureable in-vivo by assessing the retinal nerve fiber layer (RNFL) thickness in the peripapillary region and the ganglion cell layer (GCL) in the macular region by spectral-domain optical coherence tomography (SD-OCT), which enables imaging with nearly histologic resolution [3]. The only so far known treatment option with proven efficacy to decelerate disease progression in glaucoma is intraocular pressure (IOP) reduction using medications, laser and / or surgery [4].

Trabeculectomy (TE) was first described by Sugar and Cairns in the 1960s [5, 6]. TE functions through subconjunctical / sub Tenon’s drainage of aqueous humor from the anterior chamber via a trans-trabecular / trans-scleral fistulation. Even the highest IOP levels can be reduced sufficiently by TE, since the drainage channel can be adapted corresponding to visible fistulation during surgery [7]. Postsurgical stabilization of visual field indices as measured using standard automated perimetry (SAP) and deceleration of RNFL loss as measured using SD-OCT has been established for TE in recent years [8, 9].

To reduce the risk for complications in glaucoma surgery, deep sclerectomy (DS) was developed in the 1980s [10, 11]. DS differs from TE by leaving the trabecular meshwork intact and creating an intrascleral reservoir into which aqueous humor then percolates [12]. In recent years, less invasive IOP-lowering procedures have been developed, which are summarized under the term of minimally invasive glaucoma surgery (MIGS). Some MIGS comprise stent implantation utilizing subconjunctival / sub-Tenon`s drainage of aqueous humor in a comparable manner to TE and DS. The XEN microstent (XEN) (AbbVie Inc., Chicago, IL, USA) and the PRESERFLO microshunt (PF) (Glaukos Corporation, San Clemente, CA, USA) are two of these techniques [13, 14].

It has been shown before that IOP can be reduced by DS, XEN or PRESERFLO implantation comparatively effective to TE in the short and medium term after surgery [15–17]. Detailed evidence for the anatomical benefits of surgery, such as for TE, is still lacking. Therefore, the aim of this study was to investigate the postoperative development of RNFL thickness after DS, XEN and PF and to compare these results to a group of eyes in which TE was performed.

## Methods

All procedures were in accordance with the Declaration of Helsinki. Informed consent for participants was waived due to the retrospective nature of this study. The study was approved by the Ethics Commission of the Canton of Bern (BASEC-ID: ID 2022 − 01046 date of approval: 15.03.2023). Decompensated IOP, disease progression under full bearable IOP lowering medication and / or intolerance to escalation of medical therapy were the usual reasons for surgical intervention. Due to the retrospective nature of this study the indication to perform TE, DS, XEN microstent implantation or PRESERFLO microshunt implantation was dependent on the surgeons’ preferences and experiences and did not follow a clearly defined decision algorithm.

All procedures followed already published techniques [18–22]. TE, DS and PF were performed in a fornix based conjunctival flap approach. Mitomycin C (0.2 mg/ml) was applied for 2 min using surgical sponges to the scleral bed and then rinsed with 50 ml of BSS. During TE, the scleral flap was 4 × 4 mm in size, while the flap utilized in DS was 5 × 5 mm in size. In the PF cases an intrascleral channel ending in the anterior chamber angle starting 3 mm posteriorly from the corneal limbus was fashioned using a 25G angled cannula before insertion of the PF device. After preparation of the Trabeculectomy site in TE and de-roofing of Schlemm’s Canal in DS, the scleral flap was re-approximated using 2–5 non-absorbable 10/0 single button sutures. Number and tension of the flap sutures depended on the visibility of protruding aqueous humor from beneath the scleral flap. Finally, Tenon’s and conjunctiva were re-approximated to / overlapping the corneal limbus using 2 to 4 absorbable 10/0 single button sutures and 1 to 2 non-absorbable 10/0 mattress sutures to assure water tightness of the resulting bleb. XEN microstent implantation was performed in an ab interno fashion via a side port incision after filling the anterior chamber with a viscoelastic device to fashion an intrascleral tunnel of 3 mm length using the application needle. Before implantation, < 0.1 ml of Mitomycin C (0.1 mg/ml) was injected into Sub-Tenon`s space and after implantation and washing out the viscoelastic device from the anterior chamber a needling procedure was performed to free the XEN’s outer tip from adherent tissue to allow unaltered flow of aqueous humor into the preformed bleb.

Post-operative treatment regimens were similar in the different groups. Follow-up examinations were usually scheduled 1, 2 and 7 days, 2 and 3 weeks and 1, 3, 6, 12 and 24 months postoperatively. Medical treatment comprised of local antibiotics for 2 weeks (tobramycin, QID for 2 weeks), local steroids (prednisolone acetate, QID for 4 weeks then tapered by 1 drop per week) and cycloplegics (atropine 0.5%, BID for 1 week). At 1 month postoperatively, all non-absorbable and all absorbable conjunctival sutures were removed at the slit lamp. Laser suture lysis (TE, DS), needling procedures and / or 5-fluorouracil (5-FU; 0.1 ml with a concentration of 50 mg/ml) injections were considered as usual post-operative treatment modalities. Laser suture lysis and 5-FU injections were usually performed no later than 3 months after surgery. Needling procedures were performed using an operating microscope with the patient in a supine position. Laser suture lysis was performed using a Q-switched Nd: YAG laser (spot size: 100 μm, exposition time: 0.150 s., power: 200–300 mW) and a Mandelkorn suture lysis lens.

Patients had to be over 40 years of age and needed to undergo TE, DS, XEN or PF implantation for the treatment of POAG at the Bern eye clinic. The exclusion criterion was any glaucoma entity other than POAG. In cases where both eyes qualified for inclusion only the first eye undergoing surgery was analyzed, even when eyes underwent different kinds of glaucoma surgery. Demographic data such as age, sex, lens status (phakic, pseudophakic or aphakic) before surgery and laterality of the operated eye were collected. Before surgery, as well as at all scheduled follow-ups, the following examinations were performed: IOP (measured by Goldmann applanation tonometry; Haag-Streit, Köniz, Switzerland), number and frequency of applied IOP-lowering medications and best-corrected visual acuity (measured using Snellen charts with results converted to logMAR-units for statistical analysis). To be considered a surgical success, a case’s postoperative IOP had to be at least 20% lower than preoperatively and below 21 mmHg. If these criteria were met without use of IOP-lowering medications, it was considered a complete surgical success. If IOP-lowering medications were required, but the number of medications did not exceed the initial number of medications taken before surgery, it was considered a qualified success.

At 6, 12 and 24 months, an additional SD-OCT measurement (Spectralis, Heidelberg Engineering, Heidelberg, Germany) of the peripapillary RNFL (pRNFL) was performed. For segmentation of the retina / recognition of the RNFL the machines internal software tools were utilized, OCT segmentation lines were manually checked. The mean and sectoral (divided in the six Garway-Heath sectors) RNFL thickness were measured in the peripapillary oriented SD-OCT scan. A multivariable linear regression analysis was performed to identify independent predictors of pRNFL change. The covariates included age, baseline IOP, baseline pRNFL thickness, lens status and surgery type.

Data were captured and analyzed using Excel (Microsoft, Redmond, Washington, USA) and SPSS programs (IBM, Version 24.0; Chicago, Illinois, USA). Continuous variables are given as mean and standard error of mean. Categorical variables are described as frequencies. For statistical analysis Chi-square-tests, Mann–Whitney U test, non-parametric Wilcoxon- and Kruskal-Wallis-tests were performed. To account for unequal follow-up intervals, repeated measurements, and confounders, a linear mixed-effects model was performed with RNFL thickness as the outcome, surgical group, time, and their interaction as fixed effects, and baseline pRNFL, baseline IOP, age, and lens status as covariates. A first-order autoregressive (AR1) covariance structure was used to account for within-patient correlation between the two timepoints at 1 and 2 years, patients were the subject variable. Restricted maximum likelihood (REML) was used to estimate model parameters; estimated marginal means were compared pairwise between surgical groups at each timepoint; Bonferroni-adjusted contrasts were used. A p-value of less than 0.05 was considered as indicating statistical significance.

## Results

Medical records of all patients undergoing surgery between 2010 and 2020 at the Department of Ophthalmology, at Inselspital, Bern University Hospital, Switzerland were searched. 462 eyes met inclusion criteria and were added for this analysis. TE, DS, XEN microstent and PRESERFLO microshunt implantation were performed in 108, 183, 90 and 81 eyes respectively. Mean patient age was 64.2 ± 15.9, 68.9 ± 11.4, 72.4 ± 14.2 and 75.7 ± 10.9 years. 38%, 45%, 72% and 70% of cases in the TE, DS, XEN and PF groups, were pseudophakic or aphakic at the time of surgery, with a significant difference observed between the groups (*p* < 0.001, chi-square test). One eye in the TE group, two eyes in the DS group, and one eye in the XEN group were aphakic. For the sake of brevity, these were considered pseudophakic in the analysis. Subanalysis using the chi-square test revealed no significant difference in lens status between the TE and DS groups (*p* = 0.241) or the XEN and PF groups (*p* = 0.783), while confirming significant differences between all other group pairs (*p* < 0.001). Further results concerning laterality of the operated eye, gender, baseline IOP and others are reported in Table 1.

**Table 1:** Baseline demographic and clinical characteristics of the study participants

	TE (*n* = 108)	DS (*n* = 183)	XEN (*n* = 90)	PF (*n* = 81)	*p*=
Mean age; years	64.2 ± 15.9	68.9 ± 11.4	72.4 ± 14.2	75.7 ± 10.9	< 0.001 *
Laterality; right:left	47:61	95:88	54:36	36:45	
Gender; female:male	54:54	101:82	46:44	43:38	
Percentage of cases of pseudophakia or aphakia	38%	45%	72%	70%	**< 0.001** †
Baseline mean IOP; mmHg	23.7 ± 0.9	23.8 ± 0.8	21.2 ± 0.8	21.7 ± 0.8	**0.018** *
Baseline mean number of IOP-lowering medication; n	3.4 ± 0.1	3.3 ± 0.1	3.1 ± 0.2	3.5 ± 0.1	0.071 *
Baseline mean visual acuity; logMAR	0.4 ± 0.1	0.35 ± 0.04	0.53 ± 0.07	0.31 ± 0.04	0.343 *
Baseline mean pRNFL thickness; µm	64.6 ± 2.0	63.6 ± 1.8	63.2 ± 2.2	62.1 ± 1.8	0.978 *
Baseline SAP MD; dB	11.5 ± 0.9	10.6 ± 1.4	11.9 ± 1.0	11.2 ± 1.6	0.594 *

TE: trabeculectomy; DS: deep sclerectomy; XEN: XEN microstent; PF: Preserflo microshunt; IOP: intraocular pressure; pRNFL: peripapillary retinal nerve fiber layer; SAP: standard automated perimetry; MD: mean deviation. p values less than 0.05 are in bold. *: Mann–Whitney U test; †: Chi-Square test

**Fig. 1:**
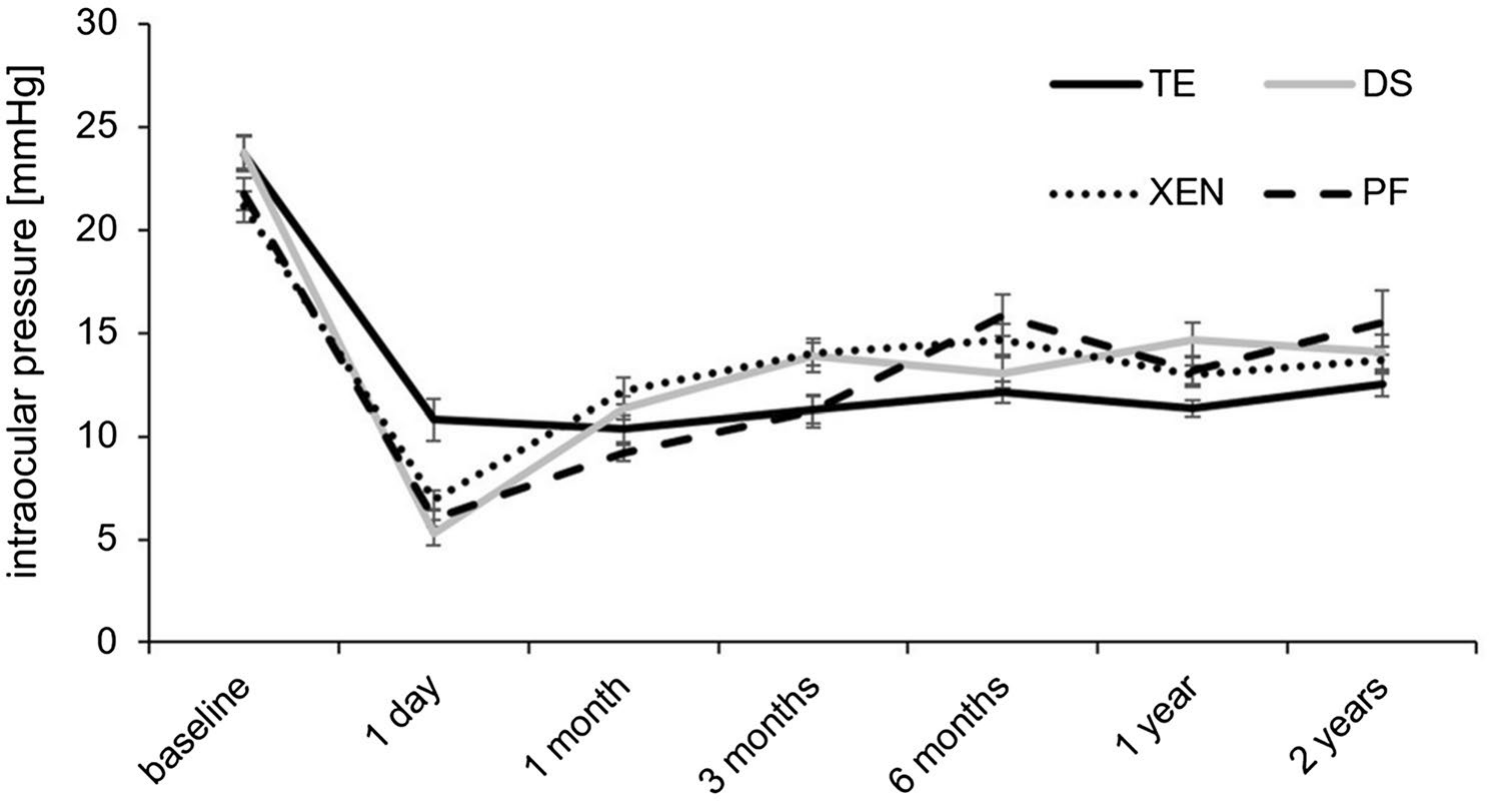
IOP results measured at baseline and during regular follow-up after TE, DS, XEN microstent or Preserflo microshunt implantation. TE: trabeculectomy; DS: deep sclerectomy; XEN: XEN microstent; PF: Preserflo microshunt

Mean IOP at baseline was higher in the TE (23.7 ± 0.9 mmHg) and DS (23.7 ± 0.8 mmHg) groups than in the XEN (21.2 ± 0.8 mmHg) and PF (21.7 ± 0.8 mmHg) groups (Table 1). Mean IOP decreased strongly in all groups, reaching 12.5 ± 0.6 mmHg (*p* < 0.001), 14.1 ± 0.9 mmHg (*p* < 0.001), 13.7 ± 0.6 mmHg (*p* < 0.001) and 15.5 ± 1.5 mmHg (*p* < 0.001) in the respective groups after 2 years (Fig. 1; Table 2). Comparison between groups showed differences of statistical significance at all follow-up time points (Table 2).

**Table 2:** Results for IOP, number of applied IOP-lowering medication and BCVA during regular follow-up after TE, DS, XEN microstent and Preserflo microshunt implantation

		TE (*n* = 108)	p= *	DS (*n* = 183)	p=*	XEN (*n* = 90)	p=*	PF (*n* = 81)	p=*	p=†
IOP [mmHg]	Baseline	23.7 ± 0.9	n.a.	23.8 ± 0.8	n.a.	21.2 ± 0.8	n.a.	21.7 ± 0.8	n.a.	0.018
	3 months	11.3 ± 0.6	< 0.001	13.9 ± 0.8	< 0.001	14.0 ± 0.5	< 0.001	11.2 ± 0.8	< 0.001	< 0.001
	6 months	12.1 ± 0.5	< 0.001	13.0 ± 0.8	< 0.001	14.7 ± 0.8	< 0.001	15.8 ± 1.0	< 0.001	< 0.001
	12 months	11.3 ± 0.4	< 0.001	14.7 ± 0.9	< 0.001	13.0 ± 0.4	< 0.001	13.1 ± 0.7	< 0.001	< 0.001
	24 months	12.5 ± 0.6	< 0.001	14.1 ± 0.9	< 0.001	13.7 ± 0.6	< 0.001	15.5 ± 1.5	= 0.028	0.04
Medication [n]	Baseline	3.4 ± 0.1	n.a.	3.3 ± 0.1	n.a.	3.1 ± 0.2	n.a.	3.5 ± 0.1	n.a.	0.071
	3 months	0.1 ± 0.1	< 0.001	0.2 ± 0.1	< 0.001	0.5 ± 0.1	< 0.001	0.1 ± 0.1	< 0.001	0.012
	6 months	0.4 ± 0.1	< 0.001	0.5 ± 0.1	< 0.001	0.7 ± 0.2	< 0.001	0.5 ± 0.2	< 0.001	0.193
	12 months	0.5 ± 0.1	< 0.001	0.7 ± 0.1	< 0.001	1.0 ± 0.2	< 0.001	0.6 ± 0.2	< 0.001	0.231
	24 months	0.6 ± 0.1	< 0.001	1.1 ± 0.2	< 0.001	0.9 ± 0.2	< 0.001	1.1 ± 0.4	= 0.001	0.211
BCVA [logMAR]	Baseline	0.4 ± 0.1	n.a.	0.4 ± 0.1	n.a.	0.5 ± 0.1	n.a.	0.3 ± 0.1	n.a.	0.343
	3 months	0.4 ± 0.1	0.667	0.4 ± 0.1	0.389	0.5 ± 0.1	0.552	0.3 ± 0.1	0.574	0.714
	6 months	0.3 ± 0.1	0.445	0.4 ± 0.1	0.708	0.5 ± 0.1	0.126	0.3 ± 0.1	0.898	0.924
	12 months	0.4 ± 0.1	0.278	0.4 ± 0.1	0.120	0.5 ± 0.1	0.413	0.3 ± 0.1	0.855	0.542
	24 months	0.4 ± 0.1	0.676	0.4 ± 0.1	0.804	0.5 ± 0.1	0.839	0.4 ± 0.1	0.628	0.921

TE: trabeculectomy; DS: deep sclerectomy; XEN: XEN microstent; PF: Preserflo microshunt; IOP: intraocular pressure; BCVA: best corrected visual acuity; *: intra-group comparison to baseline values (Wilcoxon-test); †: inter-group comparison (Kruskal-Wallis-test)

**Fig. 2:**
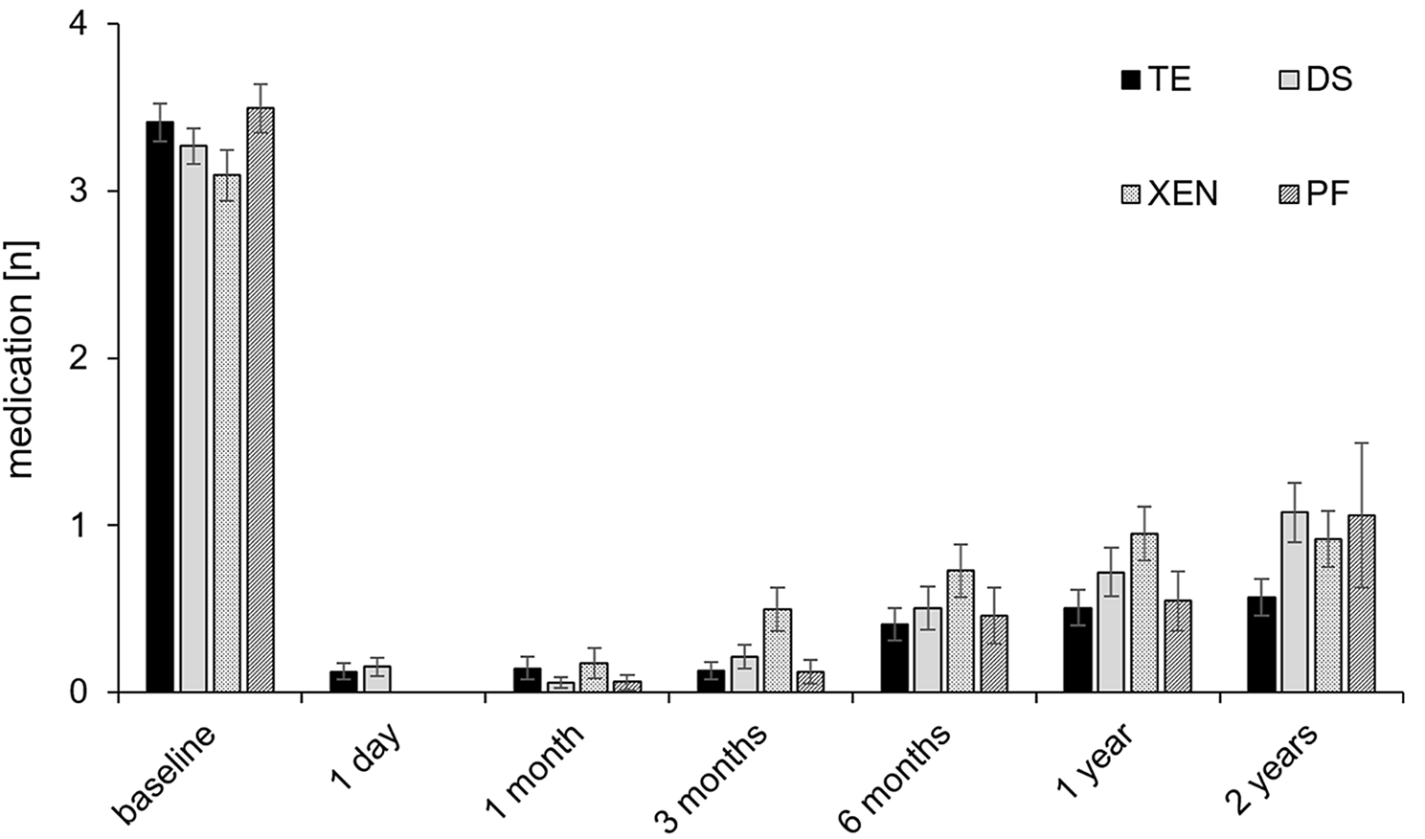
Number of IOP-lowering medication applied at baseline and during regular follow-up after TE, DS, XEN microstent or Preserflo microshunt implantation. TE: trabeculectomy; DS: deep sclerectomy; XEN: XEN microstent; PF: Preserflo microshunt

The number of IOP-lowering medications followed a comparable course as the mean IOP. Mean number of medications was 3.4 ± 0.1, 3.3 ± 0.1, 3.1 ± 0.2 and 3.5 ± 0.1 at baseline in the groups of eyes undergoing TE, DS, XEN and PF implantation (Fig. 2; Table 2). 2 years after surgery, the mean number was 0.6 ± 0.1 (*p* < 0.001), 1.1 ± 0.2 (*p* < 0.001), 0.9 ± 0.1 (*p* < 0.001) and 0.4 ± 0.1 (*p* < 0.001) in the four groups. Comparison between the four groups showed differences of statistical significance only for the follow-up three months after surgery. Further analysis showed that the number of medications at 2 years was not correlated with the change in pRNFL thickness over the same period (Pearson’s *r* = − 0.045, *p* = 0.534).

A subanalysis of pseudophakic status using the Mann–Whitney U test showed that pseudophakic patients were older, with a mean age of 74.7 ± 0.8 compared to 63.3 ± 0.9 (*p* < 0.001). Preoperative IOP was similar (22.8 ± 0.6 mmHg and 22.7 ± 0.6 mmHg, *p* = 0.981), but after 2 years, the pseudophakic group had a higher mean IOP (14.4 ± 0.7 mmHg and 13.4 ± 0.5 mmHg, *p* = 0.359). Mean medication use was higher in the pseudophakic group (baseline values: 3.5 ± 0.1 and 3.1 ± 0.1, *p* < 0.001. 2 years after surgery: 1.3 ± 0.2 and 0.7 ± 0.1, *p* = 0.004). Initially, the baseline pRNFL thickness was higher in the pseudophakic group (65.1 ± 1.3 μm vs. 61.5 ± 1.4 μm, *p* = 0.035), but after 2 years, no difference was present (59.7 ± 1.5 μm vs. 57.8 ± 1.6 μm, *p* = 0.243).

Percentage of eyes reaching complete and qualified success two years after surgery was 78% and 87% in eyes which underwent TE, 60% and 76% in the DS group, 41% and 65% in the XEN group and 55% and 65% in eyes after PRESERFLO implantation.

**Fig. 3:**
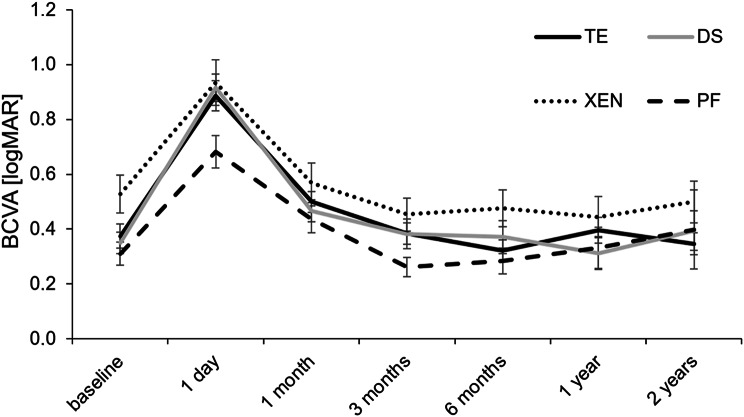
BCVA measured at baseline and during regular follow-up after TE, DS, XEN microstent or Preserflo microshunt implantation. TE: trabeculectomy; DS: deep sclerectomy; XEN: XEN microstent; PF: Preserflo microshunt

Mean BCVA remained stable (Fig. 3). Statistical analysis did not reveal differences of statistical significance between the 4 treatment groups either at baseline or at the follow-up examinations 1 and 2 years postoperatively. Intra-group comparisons between baseline and follow-up results did not show differences of statistical significance at 1 and 2 years after surgery (Table 2).

**Fig. 4:**
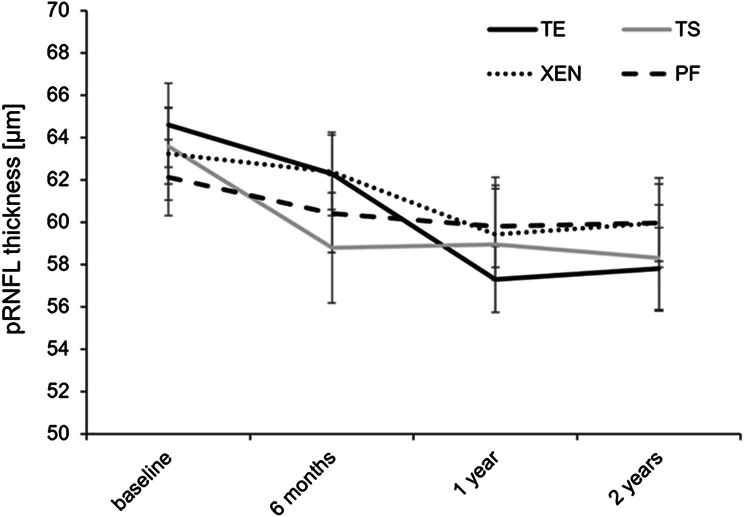
Development of mean pRNFL thickness from baseline to 2 years after surgery in eyes undergoing TE, DS, XEN microstent or Preserflo microshunt implantation. TE: trabeculectomy; DS: deep sclerectomy; XEN: XEN microstent; PF: Preserflo microshunt

Mean total pRNFL thickness declined across all treatment groups (Fig. 4). At baseline, mean pRNFL thickness was similar in every cohort: 64.6 ± 2.0 μm, 63.6 ± 1.8 μm, 63.2 ± 2.2 μm and 62.1 ± 1.8 μm in the TE, DS, XEN and PF groups (*p* = 0.978). After 2 years, mean RNFL thickness was 57.8 ± 1.9 μm, 58.3 ± 2.5 μm, 60.0 ± 2.1 μm and 59.9 ± 1.8 μm, with no statistically significant difference between groups (*p* = 0.469). pRNFL significantly decreased in each cohort at each follow-up time point except in the XEN group at 6 months (*p* = 0.183) (Table 3). Comparison of pRNFL results between baseline and follow-up results measured 1 year postoperatively revealed a difference of statistical significance in the TE (*p* < 0.001), DS (*p* = 0.016), XEN (*p* < 0.001) and PF groups (*p* = 0.009). However, a comparison of the RNFL thickness results measured at 1 and 2 years after surgery showed no further statistically significant decline of mean RNFL thickness after the first postoperative year in all 4 groups (TE: *p* = 0.131; DS: *p* = 0.374; XEN: *p* = 0.129; PF: *p* = 0.471; values not shown in Table 3). RNFL trend analyses for one representative case from each of the four surgical groups are provided in the supplementary material.

**Table 3:** Results for pRNFL thickness [µm] measured during regular follow-up after TE, DS, XEN microstent and Preserflo implantation

	TE (*n* = 108)	*p*= *	DS (*n* = 183)	*p*=*	XEN (*n* = 90)	*p*=*	PF (*n* = 81)	*p*=*	*p*=†
Baseline	64.6 ± 2.0	*n*.a.	63.6 ± 1.8	*n*.a.	63.2 ± 2.2	*n*.a.	62.1 ± 1.8	*n*.a.	0.978
6 months	62.3 ± 2.0	0.003	58.8 ± 2.6	0.016	62.4 ± 1.8	0.183	60.4 ± 1.8	0.030	0.479
12 months	57.3 ± 1.6	< 0.001	58.9 ± 3.2	0.016	59.4 ± 2.2	< 0.001	59.8 ± 1.9	0.009	0.736
24 months	57.8 ± 1.9	< 0.001	58.3 ± 2.5	0.005	60.0 ± 2.1	0.008	59.9 ± 1.8	0.008	0.469

TE: trabeculectomy; DS: deep sclerectomy; XEN: XEN microstent; PF: Preserflo microshunt; pRNFL: peripapillary retinal nerve fiber layer; *: intra-group comparison to baseline values (Wilcoxon-test); †: inter-group comparison (Kruskal-Wallis-test)

To rule out the influence of the resulting postoperative IOP on the pRNFL development, the above-described statistical analysis was repeated with pRNFL results stratified for resulting surgical success levels at 2 years postoperatively. In the group of eyes reaching a complete surgical success and a resulting IOP of < 12 mmHg at the follow-up examination 2 years after surgery, mean general pRNFL declined in a comparable manner as in the complete group of eyes described above. In these groups mean pRNFL decreased from 63.6 ± 2.3 μm to 56.4 ± 2.2 μm in the TE group (*p* = 0.002), from 62.4 ± 2.2 μm to 56.8 ± 3.5 μm in the DS group (*p* = 0.003), from 62.2 ± 2.0 μm to 57.8 ± 1.8 μm in the XEN group (*p* = 0.012) and from 63.5 ± 3.2 μm to 60.8 ± 2.9 μm in the PF group (*p* = 0.013).

**Fig. 5:**
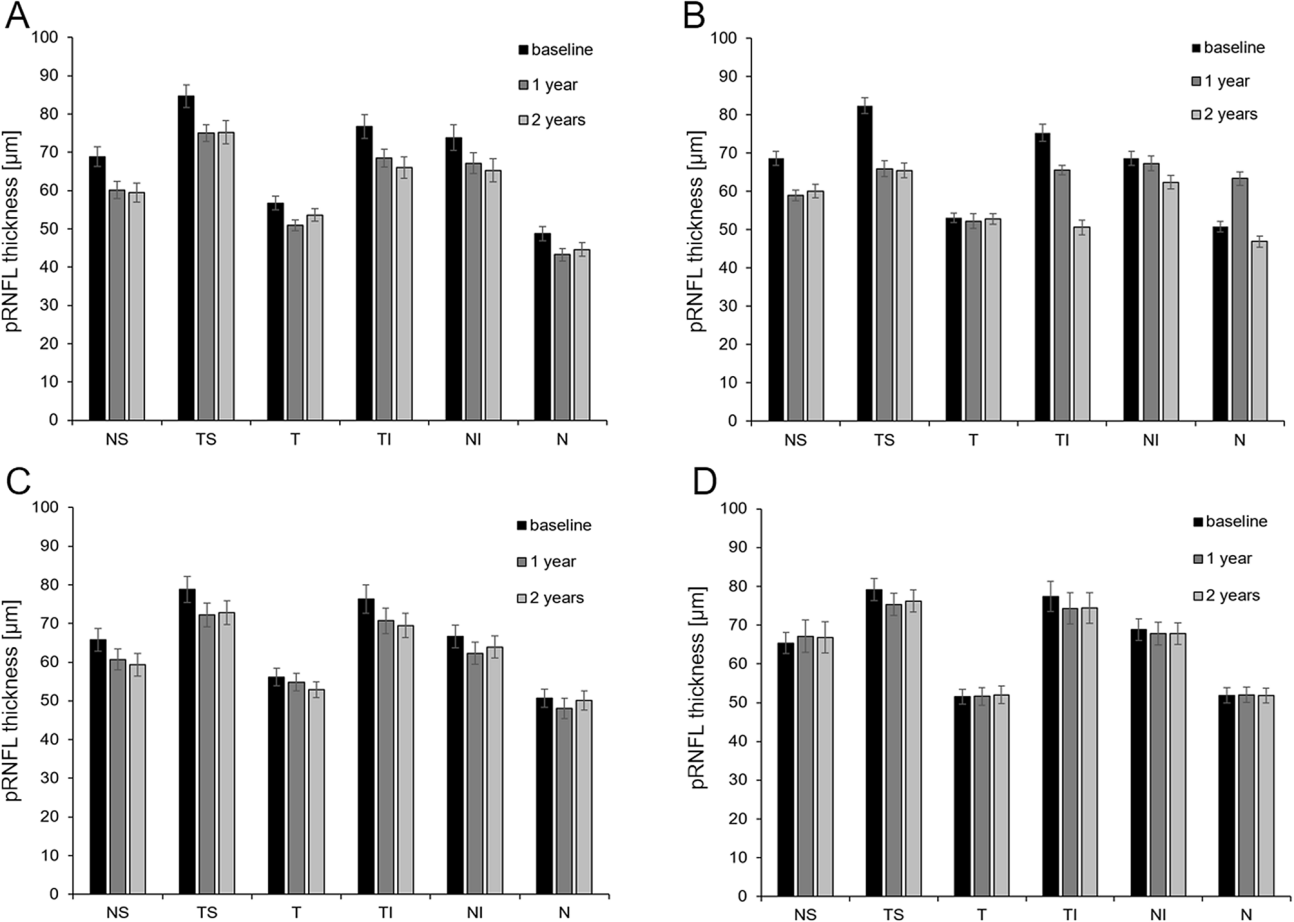
Development of mean pRNFL thickness in the six Garway-Heath sectors from baseline to 1 and 2 years after surgery in eyes undergoing TE (**A**), DS (**B**), XEN microstent (**C**) or Preserflo microshunt (**D**) implantation. TE: trabeculectomy; DS: deep sclerectomy; XEN: XEN microstent; PF: Preserflo microshunt. NS: nasal-superior; TS: temporal-superior; T: temporal; TI: temporal-inferior; NI: nasal-inferior; N: nasal

To identify which regions of the optic nerve head were most susceptible to further pRNFL decrease after surgical IOP reduction, pRNFL results for the six Garway-Heath sectors were analyzed after TE (Fig. 5). Results measured 1 and 2 years postoperatively were compared to baseline values (Tables 4 and 5). The temporal-inferior and temporal-superior Garway-Heath sectors were the sectors with the most pronounced decline in all four treatment groups.

**Table 4:** Results for pRNFL thickness [µm] in the six Garway-Heath sectors at baseline as well as 12 and 24 months after TE, DS, XEN microstent and Preserflo microshunt implantation

		NS	TS	T	TI	NI	N
TE (*n* = 108)	Baseline	68.8 ± 2.6	84.7 ± 2.9	56.8 ± 1.8	76.7 ± 3.2	73.9 ± 3.3	48.8 ± 1.9
	12 months	60.2 ± 2.2	75.1 ± 2.2	51.0 ± 1.4	68.5 ± 2.3	67.2 ± 2.8	43.3 ± 1.6
	24 months	59.5 ± 2.5	75.3 ± 3.0	53.6 ± 1.6	66.0 ± 2.8	65.3 ± 3.1	44.6 ± 1.8
DS (*n = 183)*	Baseline	68.7 ± 1.9	82.4 ± 2.1	53.1 ± 1,2	75.3 ± 2.2	68.6 ± 1.8	50.8 ± 1.4
	12 months	58.9 ± 1.4	65.9 ± 2.1	52.2 ± 2.0	65.6 ± 1.2	67.3 ± 2.0	63.4 ± 1.8
	24 months	60.0 ± 1.8	65.4 ± 1.9	52.8 ± 1.4	50.6 ± 1.9	62.4 ± 1.7	46.9 ± 1.5
XEN (*n* = 90)	Baseline	65.7 ± 2.9	78.8 ± 3.4	56.2 ± 2.3	76.3 ± 3.7	66.6 ± 3.0	50.7 ± 2.4
	12 months	60.7 ± 2.7	72.3 ± 3.1	54.8 ± 2.3	70.8 ± 3.3	62.4 ± 2.8	48.1 ± 2.7
	24 months	59.3 ± 3.0	72.8 ± 3.1	52.9 ± 2.1	69.5 ± 3.2	63.9 ± 2.9	50.1 ± 2.5
PF (*n* = 81)	Baseline	65.3 ± 2.7	79.2 ± 2.9	51.5 ± 2.0	77.4 ± 3.9	68.8 ± 2.7	51.8 ± 2.0
	12 months	67.1 ± 4.2	75.4 ± 2.9	51.7 ± 2.3	74.4 ± 4.0	67.8 ± 2.9	52.1 ± 2.0
	24 months	66.9 ± 4.0	76.3 ± 2.9	52.0 ± 2.3	74.4 ± 4.0	67.8 ± 2.8	51.8 ± 1.9

TE: trabeculectomy; DS: deep sclerectomy; XEN: XEN microstent; PF: Preserflo microshunt; NS: nasal-superior; TS: temporal-superior; T: temporal; TI: temporal-inferior; NI: nasal-inferior; N: nasal

**Table 5:** Statistical analysis of pRNFL thicknesses [µm] results measured in the six Garway-Heath sectors after TE, DS, XEN microstent and Preserflo microshunt implantation. Comparison between baseline results and results measured 12 and 24 months after surgery using non-parametric Wilcoxon-test

		NS	TS	T	TI	NI	N
TE (*n* = 108)	12 months	**< 0.001**	**< 0.001**	**0.003**	**0.004**	**< 0.001**	**0.008**
	24 months	0.587	0.141	0.300	0.315	0.555	0.765
DS (*n* = 183)	12 months	**0.016**	**< 0.001**	0.061	**0.027**	0.647	**0.013**
	24 months	0.349	0.871	0.373	0.410	0.374	0.785
XEN (*n* = 90)	12 months	**0.027**	**< 0.001**	**0.006**	**< 0.001**	**< 0.001**	0.126
	24 months	0.183	0.652	0.141	0.069	0.934	0.671
PF (*n* = 81)	12 months	0.413	**0.011**	0.403	**0.016**	0.203	0.567
	24 months	0.286	0.138	0.082	0.722	0.721	0.822

TE: trabeculectomy; DS: deep sclerectomy; XEN: XEN microstent; PF: Preserflo microshunt; NS: nasal-superior; TS: temporal-superior; T: temporal; TI: temporal-inferior; NI: nasal-inferior; N: nasal

Multivariable linear regression analysis revealed that greater baseline pRNFL thickness (B = 0.238, *p* < 0.001) and higher baseline IOP (B = 0.177, *p* = 0.046) were independently associated with increased pRNFL thinning. However, surgery type (*p* = 0.175), age (*p* = 0.895) and lens status (*p* = 0.405) were not significantly associated with pRNFL change. IOP reduction showed a weak but significant positive correlation with RNFL thinning (*r* = 0.165, *p* = 0.030), indicating that greater pressure reduction was associated with increased thinning. In contrast, postoperative IOP at two years was not significantly associated with RNFL thinning (*r* = − 0.033, *p* = 0.664). These findings could be compatible with a contribution of pressure-related structural remodeling to postoperative pRNFL thinning; however, continued glaucomatous progression, delayed manifestation of preoperative axonal injury, or a combination of these mechanisms are also a possible explanation.

In the linear mixed-effects model (*n* = 263 with complete covariate data), there was no significant Time × Group interaction (F(3, 203.6) = 0.82, *p*=0.484), suggesting that the pattern of RNFL change between 1 and 2 years postoperatively did not differ between surgical techniques. Neither the main effect of Time (F(1, 205.4) = 0.64, *p*=0.424) nor Group (F(3, 247.6) = 1.38, *p*=0.250) was significant. Baseline pRNFL was the only significant predictor of RNFL thickness changes (F(1, 248.1) = 504.35, *p*<0.001).

## Discussion

RNFL decline continues during the first year after glaucoma surgery and IOP normalization even in cases where very low IOP-levels of 12 mmHg and lower were reached. Comparison between RNFL thickness results measured at baseline as well as 1 and 2 years after surgery revealed differences of statistical significance after all four included surgical IOP-lowering techniques (TE, DS, XEN and PF). RNFL sector analysis of the six Garway-Heath sectors showed that postoperative decline is most pronounced in the temporal sectors of the optic nerve head. Further RNFL decline and higher susceptibility of the temporal nerve head sectors are two important points that should be kept in mind when monitoring patients after glaucoma surgery [23].

For all here included surgical techniques (TE, DS, XEN or PF), several trials with follow-up periods of different lengths already existed before our analysis. All four techniques have been shown to lower IOP and number of IOP-lowering medications with high percentages of surgical success [24–28]. Edmunds et al. [24] in the 1990s and Kirwan et al. [25] in the early 2000s demonstrated, using results from > 1,200 and > 400 eyes, that TE led to a mean IOP decrease from 26.2 to 14.4 mmHg and from 23.0 to 12.4 mmHg while medication burden could be reduced effectively. The APEX multicenter trial [26] demonstrated an effective decrease of mean IOP from 21.4 ± 3.6 to 15.2 ± 4.2 mmHg and a decrease of the number of the prescribed IOP-lowering medication from 2.7 ± 0.9 to 1.1 ± 1.2 over a mean follow-up of 24 months after implantation of the XEN microstent in POAG eyes. Equally, it was demonstrated using the results from a large multicenter trial by Beckers et al. that implantation of the PF microshunt lowers IOP from a mean of 21.7 ± 3.4 mmHg to 14.5 ± 4.6 mmHg and the number of IOP-lowering medication from 2.1 ± 1.3 to 0.5 ± 0.9 efficiently and durably over a medium-term follow-up of 24 months [27].

We demonstrated a strong reduction of IOP and IOP-lowering medication in all four groups undergoing TE, DS, XEN or PF implantation over a follow-up of 24 months. However, we found a statistically significant continuing decline of RNFL thickness during the first year postoperatively in all four groups. SD-OCT measurements showed a mean RNFL decline of 10.5%, 8.3%, 5.1% and 3.5% µm in the TE, DS, XEN and PF groups during the 2 years of follow-up. Of major interest to us was also the location of further postsurgical RNFL decline at the superior and inferior poles of the optic nerve head. These are also the two typical locations for development of RNFL decline during the natural course in glaucoma [23].

RNFL decline progressed during the first postoperative year, while differences between measurements 1 and 2 years after the respective surgery did not reach statistical significance. This is an observed finding, while the underlying reasons remain speculative. It might be hypothesized that continuous postsurgical RNFL decline during the first year after surgery is due to the continued glaucoma progression leading to RGC demise until surgical intervention and IOP normalization and that preoperative axon loss still manifests after surgery in a delayed manner, taking until 6 to 12 months postoperatively to develop completely. On the other hand, induced surgical trauma might also have an impact on pRNFL thinning, and structural remodeling of the optic nerve head or segmentation-related measurement changes might also contribute. None of these mechanisms were directly assessed and should therefore be regarded as hypothetical.

The finding of ongoing RNFL decline after surgical IOP-reduction is not novel. Chua and colleagues [28] demonstrated a mean pRNFL decrease of -4.21 μm/year in a group of 130 eyes during the first year after TE. This is comparable to our analyzed TE and DS cohorts. Comparatively, Demirtas and colleagues [29], analyzing the post-surgical development in 32 POAG eyes undergoing TE, found a mean RNFL decrease of 9.6 ± 14.4 μm (*p* = 0.002) during the first year after surgery. pRNFL results measured at the 2nd (*p* = 0.06) and 3rd (*p* = 0.88) year after TE did not show statistical significance when compared to the results obtained 1 year postoperatively. This is also comparable to our results describing a deceleration of pRNFL decline 1 year postoperatively.

The secondary analysis revealed higher rates of pseudophakic eyes in the XEN and PF groups. The following explanations are proposed rather than directly tested. This may be due to the older age of the patients in these two groups, especially given that the pseudophakic eyes were older. MIGS tend to be performed on older patients [30], which may have influenced these results. Higher IOP may also be due to the advanced age and consequently disease progression of pseudophakic patients [31]. This may have led to poorer medication outcomes.

These findings were further analyzed by a linear mixed-effects model accounting for repeated measurements and confounders: RNFL changes between 1 and 2 years did not differ significantly between cohorts; baseline structural status was the main determinant of follow-up RNFL thickness. This strengthens our original observations: the plateau observed in the first postoperative year is consistent across all groups rather than being driven by any single cohort.

These findings have an impact on counselling of patients after glaucoma surgery. Firstly, the existence of ongoing further RNFL decline after surgery should be known to the treating physician and should be weighed accordingly in view of a surgical success clinically defined by reduction of IOP and IOP-lowering medication. Therefore, clinicians should keep in mind that pRNFL thinning can continue through the first postoperative year, even with good IOP control. This alone should not be interpreted as treatment failure. At the same time, we should exercise caution when factoring this early decline into judgments of long-term progression since we cannot yet fully explain why it occurs. Treating it too readily as expected or benign could result in missing genuine ongoing damage. Secondly, in view of the mean pRNFL and SAP results before surgery in all four groups, which reflect advanced-stage glaucoma cases, it might be argued that surgical intervention should have been scheduled earlier in the disease course. The growing availability of MIGS might prove helpful in this regard.

Our study is certainly not without the expected weaknesses of a retrospective, clinical study. Although this study comprises a large cohort of glaucoma patients, longer follow-up periods and a multicenter and double-blinded study design would be desirable. A longitudinal structure–function correlation was not performed, as SAP was not tested at standardized intervals across the full cohort. Without such data, we cannot determine whether the observed RNFL decline corresponds to functional disease progression, anatomical remodeling, or measurement variability. Prospective studies including serial visual field testing alongside OCT are needed to resolve this question. Additionally, this study relied exclusively on pRNFL measurements. As IOP reduction may alter optic nerve head configuration and lamina cribrosa biomechanics, macular GCIPL (ganglion cell–inner plexiform layer) or GCC (Ganglion Cell Complex) measurements, which were not systematically analyzed in our cohort, would help to further evaluate postoperative structural change and should be incorporated in future studies. The cohorts differed significantly in age, lens status and baseline IOP, reflecting the non-randomized allocation inherent to this retrospective design. Although we adjusted for these covariates in the multivariable regression, residual confounding cannot be excluded, and between-group comparisons should be interpreted considering this limitation. Moreover, the study population predominantly consisted of patients with mid-stage to advanced glaucoma. This may have influenced the study outcomes due to the floor effect in the pRNFL analysis. This is particularly pertinent given that greater baseline pRNFL thickness and higher baseline IOP were independently associated with increased RNFL thinning. However, the postoperative OCT development and comparison between different types of glaucoma interventions are, in our opinion, well worth reporting.

We reported the development of mean RNFL during 2 years of follow-up after successful TE, DS, XEN microstent and PRESERFLO microshunt implantation. We showed that although IOP and IOP-lowering medication were successfully reduced, RNFL further declines during the first year, with no further statistically significant decline observed during the second postoperative year. These findings should be kept in mind when counselling glaucoma patients after surgery and evaluating surgical success and further interventions.

## Supplementary Information

Below is the link to the electronic supplementary material.

Supplementary Material 1

## Data Availability

The datasets used for this study are available from the corresponding author upon reasonable request.

## Abbreviations

BCVA: Best-corrected visual acuity
DS: Deep sclerectomy
GCC: Ganglion cell complex
GCIPL: Ganglion cell–inner plexiform layer
GCL: Ganglion cell layer
IOP: Intraocular pressure
logMAR: Logarithm of the minimum angle of resolution
MD: Mean deviation
MIGS: Minimally invasive glaucoma surgery
PF: Preserflo microshunt
POAG: Primary open-angle glaucoma
pRNFL: Peripapillary retinal nerve fiber layer
RGC: Retinal ganglion cell
RNFL: Retinal nerve fiber layer
SAP: Standard automated perimetry
SD-OCT: Spectral-domain optical coherence tomography
TE: Trabeculectomy
XEN: XEN microstent

## References

[1] Quigley HA. Neuronal death in glaucoma. Prog Retin Eye Res. 1999;18(1):39–57. 9920498 10.1016/s1350-9462(98)00014-7

[2] Harwerth RS, Quigley HA. Visual field defects and retinal ganglion cell losses in patients with glaucoma. Arch Ophthalmol. 2006;124(6):853–9. 16769839 10.1001/archopht.124.6.853 PMC2265071

[3] Smith C, Vianna J, Chauhan B. Assessing retinal ganglion cell damage. Eye (Lond). 2017;31:209–17. 28085141 10.1038/eye.2016.295 PMC5306472

[4] Weinreb RN, Aung T, Medeiros FA. The pathophysiology and treatment of glaucoma: a review. JAMA. 2014;311(18):1901–11. 24825645 10.1001/jama.2014.3192 PMC4523637

[5] Sugar HS. Experimental trabeculectomy in glaucoma. Am J Ophthalmol. 1961;51:623–7.

[6] Cairns JE. Trabeculectomy: preliminary report of a new method. Am J Ophthalmol. 1968;66:673–9. 4891876

[7] Razeghinejad MR, Fudemberg SJ, Spaeth GL. The changing conceptual basis of trabeculectomy: a review of past and current surgical techniques. Surv Ophthalmol. 2012;57(1):1–25. 22137574 10.1016/j.survophthal.2011.07.005

[8] Aachal K, Alexander S, Catey B, et al. Optic disc and visual field changes after trabeculectomy. Invest Ophthalmol Vis Sci. 2009;50(10):4693–9. 19474409 10.1167/iovs.08-3115

[9] Schargus M, Busch C, Rehak M, et al. Functional monitoring after trabeculectomy or XEN Microstent implantation using spectral-domain optical coherence tomography and visual field indices—a retrospective comparative cohort study. Biology (Basel). 2021;10(4):273. 33801601 10.3390/biology10040273 PMC8065996

[10] Netland PA, Ophthalmic Technology Assessment Committee Glaucoma Panel. American Academy of Ophthalmology. Nonpenetrating glaucoma surgery. Ophthalmology. 2001;108(2):416–21. 11158823 10.1016/s0161-6420(00)00647-3

[11] Klemm M. Tiefe Sklerektomie. Eine Alternative zur Trabekulektomie [Deep sclerectomy. An alternative to trabeculectomy]. Ophthalmologe. 2015;112(4):313–8. 25783165 10.1007/s00347-014-3161-6

[12] Richardson-May J, Alnuaimi R, Elbably A, et al. Our experience of deep sclerectomy at a tertiary center in the United Kingdom over 14 years. Cureus. 2023;15(8):e43366. 37701011 10.7759/cureus.43366 PMC10494555

[13] Fea AM, Durr GM, Marolo P, et al. XEN^®^ Gel Stent: a comprehensive review on its use as a treatment option for refractory glaucoma. Clin Ophthalmol. 2020;14:1805–32. 32636610 10.2147/OPTH.S178348 PMC7335291

[14] Saeed E, Gołaszewska K, Dmuchowska DA, et al. The PreserFlo MicroShunt in the context of minimally invasive glaucoma surgery: a narrative review. Int J Environ Res Public Health. 2023;20(4):2904. 36833599 10.3390/ijerph20042904 PMC9957246

[15] Elhofi A, Helaly HA. Non-penetrating deep sclerectomy versus trabeculectomy in primary congenital glaucoma. Clin Ophthalmol. 2020;14:1277–85. 32494118 10.2147/OPTH.S253689 PMC7229790

[16] Sheybani A, Vera V, Grover DS, et al. Gel stent versus trabeculectomy: the randomized, multicenter, Gold-Standard Pathway Study (GPS) of effectiveness and safety at 12 months. Am J Ophthalmol. 2023;252:306–25. 36972738 10.1016/j.ajo.2023.03.026

[17] Zweifel LAB, Storp JJ, Vietmeier FE, et al. PreserFlo MicroShunt versus trabeculectomy: efficacy and surgical success within a heterogenous patient cohort. Life (Basel). 2024;14(9):1171. 39337954 10.3390/life14091171 PMC11433034

[18] Jones E, Clarke J, Khaw PT. Recent advances in trabeculectomy technique. Curr Opin Ophthalmol. 2005;16(2):107–13. 15744141 10.1097/01.icu.0000156138.05323.6f

[19] Mermoud A, Schnyder CC. Nonpenetrating filtering surgery in glaucoma. Curr Opin Ophthalmol. 2000;11(2):151–7. 10848223 10.1097/00055735-200004000-00015

[20] Anand N, Kumar A, Gupta A. Primary phakic deep sclerectomy augmented with mitomycin C: long-term outcomes. J Glaucoma. 2011;20(1):21–7. 20179623 10.1097/IJG.0b013e3181ccb926

[21] Gambini G, Carlà MM, Giannuzzi F, et al. PreserFlo^®^ MicroShunt: an overview of this minimally invasive device for open-angle glaucoma. Vis (Basel). 2022;6(1):12. 10.3390/vision6010012 PMC8883991 35225971

[22] Linton E, Au L. Technique of XEN implant revision surgery and the surgical outcomes: a retrospective interventional case series. Ophthalmol Ther. 2020;9(1):149–57. 32062789 10.1007/s40123-020-00234-0 PMC7054468

[23] Baniasadi N, Paschalis EI, Haghzadeh M, et al. Patterns of retinal nerve fiber layer loss in different subtypes of open-angle glaucoma using spectral-domain optical coherence tomography. J Glaucoma. 2016;25(10):865–72. 27599175 10.1097/IJG.0000000000000534

[24] Edmunds B, Thompson J, Salmon J, et al. The National Survey of Trabeculectomy. II. Variations in operative technique and outcome. Eye (Lond). 2001;15:441–8. 11767016 10.1038/eye.2001.152

[25] Kirwan JF, Lockwood AJ, Shah P, Trabeculectomy Outcomes Group Audit Study Group, et al. Trabeculectomy in the 21st century: a multicenter analysis. Ophthalmology. 2013;120(12):2532–9. 24070811 10.1016/j.ophtha.2013.07.049

[26] Reitsamer H, Sng C, Vera V, et al. Two-year results of a multicenter study of the ab interno gelatin implant in medically uncontrolled primary open-angle glaucoma. Graefes Arch Clin Exp Ophthalmol. 2019;257(5):983–96. 30758653 10.1007/s00417-019-04251-z

[27] Beckers HJM, Aptel F, Webers CAB, et al. Safety and effectiveness of the PRESERFLO^®^ MicroShunt in primary open-angle glaucoma: results from a 2-year multicenter study. Ophthalmol Glaucoma. 2022;5(2):195–209. 34329772 10.1016/j.ogla.2021.07.008

[28] Chua J, Kadziauskienė A, Wong D, et al. One-year structural and functional glaucoma progression after trabeculectomy. Sci Rep. 2020;10(1):2808. 32071369 10.1038/s41598-020-59792-9 PMC7029027

[29] Demirtaş AA, Karahan M, Erdem S, et al. Long-term effects of trabeculectomy in primary open-angle glaucoma on segmented macular ganglion cell complex alterations. Int Ophthalmol. 2021;41(6):2249–63. 33880684 10.1007/s10792-021-01840-y

[30] Olivier MMG, Smith OU, Croteau-Chonka CC, et al. Demographic and clinical characteristics associated with minimally invasive glaucoma surgery use: an Intelligent Research in Sight (IRIS^®^) Registry retrospective cohort analysis. Ophthalmology. 2021;128(9):1292–9. 33600867 10.1016/j.ophtha.2021.02.012

[31] Chan MPY, Grossi CM, Khawaja AP, et al. Associations with intraocular pressure in a large cohort: results from the UK Biobank. Ophthalmology. 2016;123(4):771–82. 26795295 10.1016/j.ophtha.2015.11.031 PMC4819446

