# Supplementary material for: Longitudinal OCT changes of the peripapillary RNFL after different glaucoma interventions – a comparative study
Source: BMC Ophthalmol. 2026 Jul 31;26:456. doi: 10.1186/s12886-026-05160-6 (PMC13430828; doi:10.1186/s12886-026-05160-6)

**Supplementary material:**

Supplementary Figure 1: RNFL trend analysis of one Trabeculectomy case.


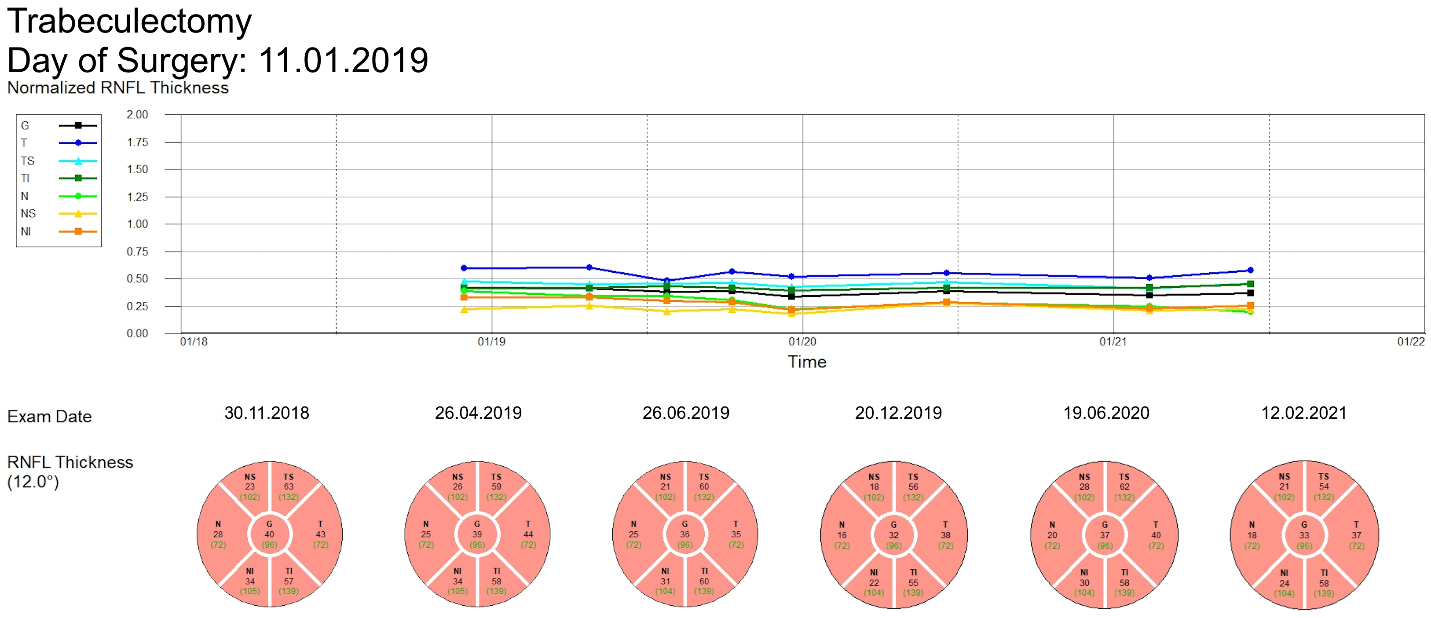


Supplementary Figure 2: RNFL trend analysis of one Deep Sclerectomy case.


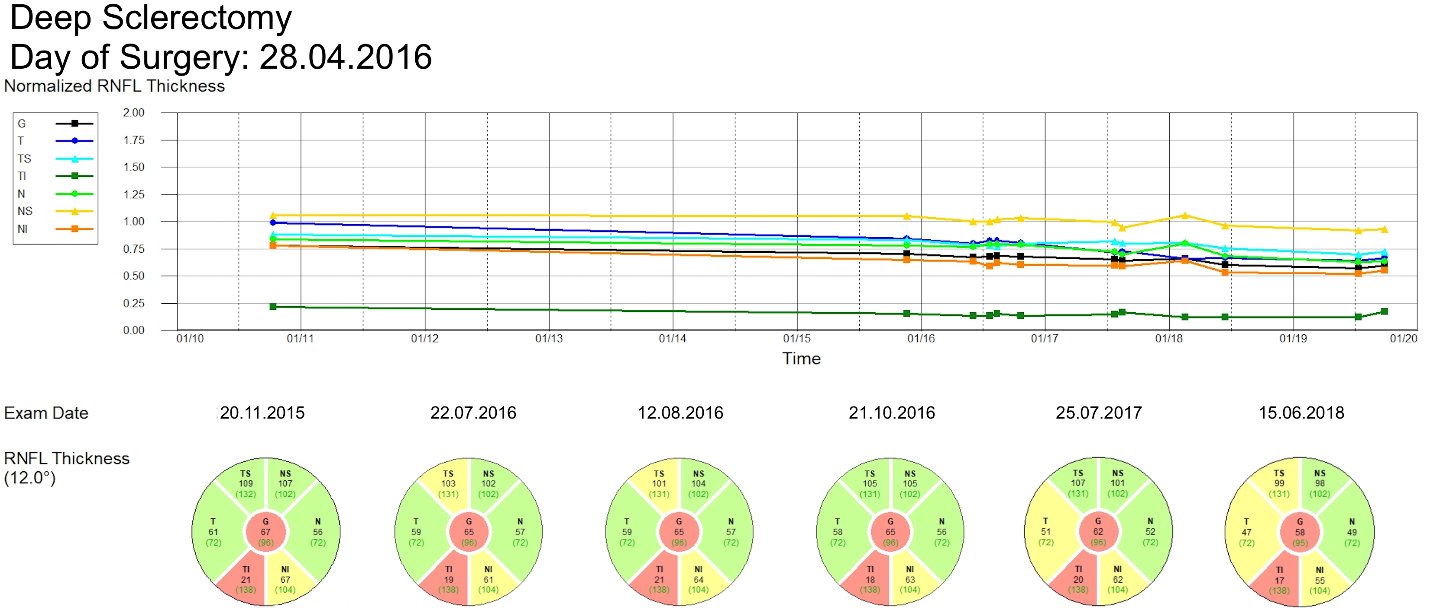


Supplementary Figure 3: RNFL trend analysis of one XEN Microstent case.


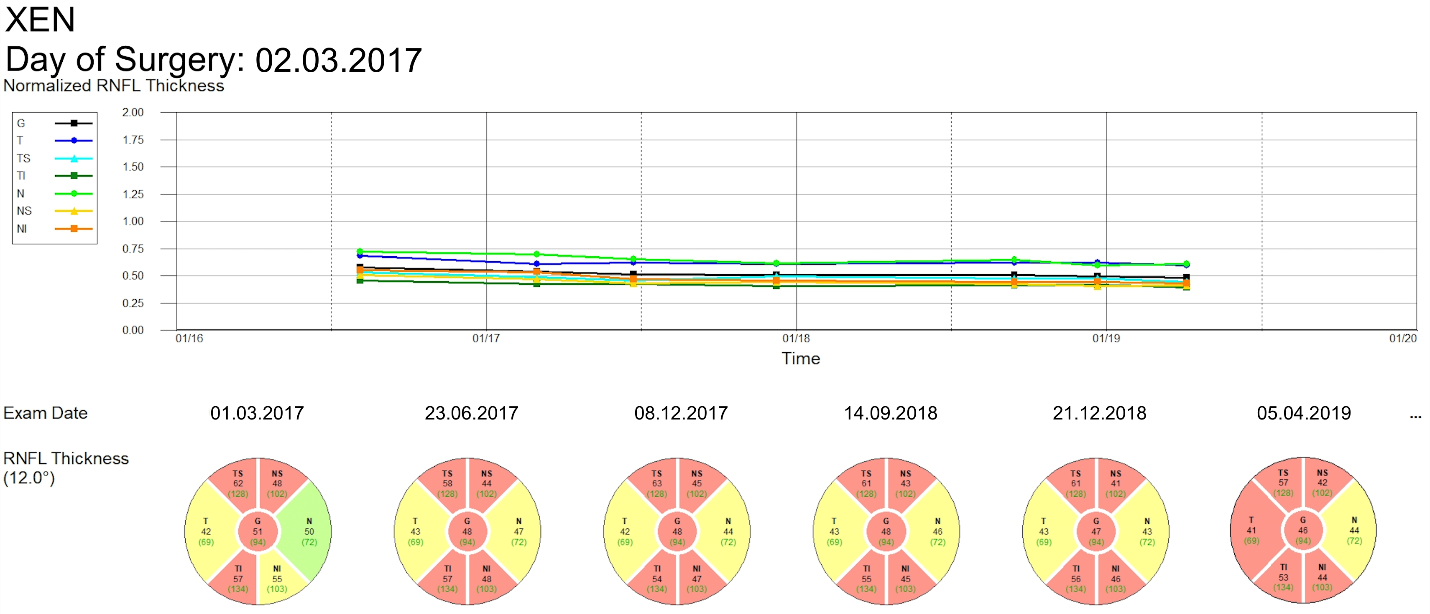


Supplementary Figure 4: RNFL trend analysis of one Preserflo Microshunt case.


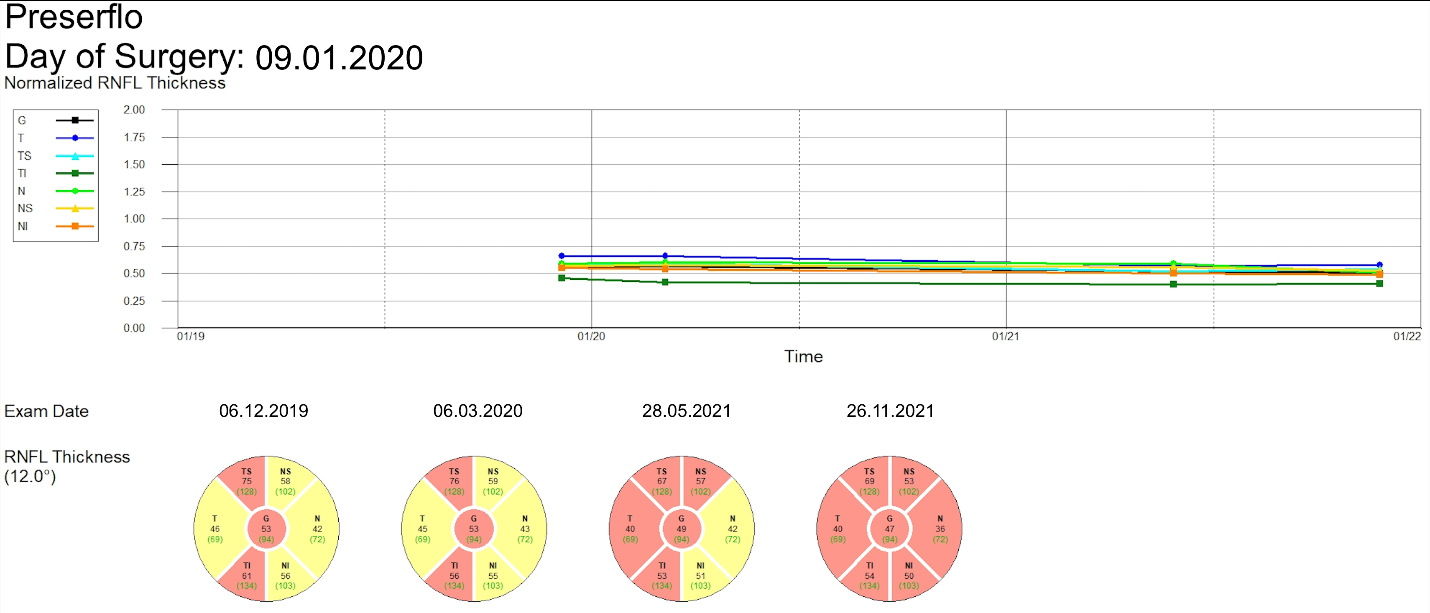

Supplement: Supplementary file 1 — Supplementary Material 1 [file 12886_2026_5160_MOESM1_ESM.docx]
